# Who uses outpatient healthcare services under Ghana’s health protection scheme and why?

**DOI:** 10.1186/s12913-016-1429-z

**Published:** 2016-05-10

**Authors:** Ama P. Fenny, Felix A. Asante, Daniel K. Arhinful, Anthony Kusi, Divya Parmar, Gemma Williams

**Affiliations:** Economics Division, Institute of Statistical, Social and Economic Research (ISSER), University of Ghana, P.O. Box LG 74, Legon, LG74 Accra Ghana; Department of Epidemiology, Noguchi Memorial Institute for Medical Research, University of Ghana, LG 581, Legon, Accra Ghana; School of Health Sciences, City University London, Northampton Square, London, EC1V 0HB UK; LSE Health, London School of Economics and Political Science, Houghton Street, 19, London, WC2A 2AE UK

**Keywords:** Utilisation, National Health Insurance, Ghana, Outpatient services

## Abstract

**Background:**

The National Health Insurance Scheme (NHIS) was launched in Ghana in 2003 with the main objective of increasing utilisation to healthcare by making healthcare more affordable. Previous studies on the NHIS have repeatedly highlighted that cost of premiums is one of the major barriers for enrollment. However, despite introducing premium exemptions for pregnant women, older people, children and indigents, many Ghanaians are still not active members of the NHIS. In this paper we investigate why there is limited success of the NHIS in improving access to healthcare in Ghana and whether social exclusion could be one of the limiting barriers. The study explores this by looking at the Social, Political, Economic and Cultural (SPEC) dimensions of social exclusion.

**Methods:**

Using logistic regression, the study investigates the determinants of health service utilisation using SPEC variables including other variables. Data was collected from 4050 representative households in five districts in Ghana covering the 3 ecological zones (coastal, forest and savannah) in Ghana.

**Results:**

Among 16,200 individuals who responded to the survey, 54 % were insured. Out of the 1349 who sought health care, 64 % were insured and 65 % of them had basic education and 60 % were women. The results from the logistic regressions show health insurance status, education and gender to be the three main determinants of health care utilisation. Overall, a large proportion of the insured who reported ill, sought care from formal health care providers compared to those who had never insured in the scheme.

**Conclusion:**

The paper demonstrates that the NHIS presents a workable policy tool for increasing access to healthcare through an emphasis on social health protection. However, affordability is not the only barrier for access to health services. Geographical, social, cultural, informational, political, and other barriers also come into play.

## Background

Over the past decade, a number of countries in sub-Saharan Africa have adopted social health insurance schemes, including Nigeria, Rwanda, Tanzania, and Ghana. For example, in 1999, the Government of Rwanda developed a community-based health insurance scheme which was expanded nationally in 2006. The aim was to increase financial resources for the local health-care system and to improve access to health care for vulnerable groups. Likewise, Ghana’s National Health Insurance Scheme (NHIS) was launched in 2003 with the aim of ultimately providing affordable and equitable access to basic healthcare services for the entire populace.

Social health insurance is increasingly recognised as one of the health financing approaches with a strong potential to address equity and social protection issues in healthcare especially in developing countries. By pooling risks and resources it promises to ensure better access and provide risk protection to the poor against the cost of illness [1–5]. A review by the Ghana Health Service [6] shows that since the start of the NHIS in 2005, overall outpatient department (OPD) cases have shown a marked increase, suggesting that the NHIS policy has led to an increase in health service usage.

Social health protection is a critical component of social protection, underpinned by principles of solidarity and equity. Traditional concepts of social protection, based on the notion of mutual support, are still strongly rooted in Ghanaian culture, but are eroding under the influence of modernisation. In Ghana, health inequities are seen to be a major form of social exclusion. There is striking evidence of rural-urban disparities in access to health care services, inequitable distribution of health workers; striking disparities in access to health services between rich and poor and gender gaps in access due to poverty, deprivation and ignorance. There are indications that some segments of society are not being reached by these social health protection programmes. For example, in Ghana a number of studies have shown that individuals from richer quintiles are more likely to be enrolled into the NHIS scheme than those in poorer quintiles [7–9]. Parma et al. [10] also find health insurance lowest in the poorer quartiles among the elderly in Ghana and Senegal. This invariably implies that access to healthcare is restricted to individuals who are able to afford insurance; leaving the poor and vulnerable groups without access to care. To compound these challenges, enrollment in the scheme in Ghana has fallen to a coverage of less than 40 % of the total population from about 67 % in 2010 [11].

The structures of social systems and relationships produce exclusionary processes that limit the success of social health protection programs. This study adopts the SEKN (Social Exclusion Knowledge Network) definition of social exclusion which defines it as multidimensional processes driven by unequal power relationships interact across four main dimensions—social, political, economic and cultural [12]. Based on this and an extensive literature review, a Social, Political, Economic and Cultural (SPEC) framework was developed which identifies domains and variables that allow the study to capture all aspects of social exclusion [10]. This is explained in detail in the methodology section. The paper seeks to assess the impact of NHIS on the utilisation of outpatient healthcare services in Ghana through this SPEC lens. Specifically, to determine which groups use health services and which processes explain why. The rest of the paper is structured as follows: the next section gives an overview of the health system, followed by the methodology, results and discussion sections. The final section gives a brief conclusion.

### The Ghana Health System

Healthcare delivery in Ghana is provided by both the public and private (private-for-profit and private-not-for-profit) sectors, with the public sector organised according to hierarchy with the national (teaching hospitals) at the apex, followed by regional (regional hospitals), district (district hospitals), sub-district (health centres) and community levels (CHPS). Sub-district (health centres) and community levels (CHPS) provide primary care, with district and regional hospitals providing secondary healthcare as well as primary healthcare. Tertiary services including specialised clinical care are provided at the teaching hospitals. District hospitals are staffed with one or more qualified medical doctors, nurses, pharmacists, laboratory technicians, auxiliary nurses and other support personnel. Health centres are manned by a medical assistant or a nurse.

Healthcare financing in Ghana has gone through many dynamics, from free healthcare at the eve of independence, introduction of the nominal fee in the 1970s and the 1980s full cost recovery, popularly known as the ‘Cash and Carry’ system. Recognising that direct out-of- pocket payment limited access to healthcare, the Government of Ghana declared its intention to abolish the system, and began exploring the feasibility of introducing a national health insurance scheme to be managed at the district level. The National Health Insurance Act, 2003 (Act 650) established the NHIS with the aim of increasing access to healthcare and improving the quality of basic healthcare services for all citizens, especially the poor and vulnerable. The defined benefit package under the scheme includes inpatient hospital care, outpatient care at primary and secondary levels, and emergency and transfer services. Premiums are charged to each client and are renewable on a yearly basis.

## Methods

### The SPEC (social, political, economic and cultural) framework

The SPEC framework was developed on the premise that there were a number of risk factors which contributed to an individual’s vulnerability to social exclusion [10]. The task of identifying variables of social exclusion within the four main domains was undertaken as part of the initial steps of the study. In the framework, the social dimension is constituted by proximal relationships of support and solidarity (such as friendship, kinship, family, neighbourhood, community, social movements) that generate a sense of belonging within social systems. Social bonds are strengthened or weakened along this dimension [12]. The political dimension considers power dynamics in relationships which generate unequal patterns of formal rights embedded in legislation, constitutions, policies and practices and the conditions in which rights are exercised, including access to safe water, sanitation, shelter, health services, transport and power. The economic dimension is constituted by access to, and distribution of, material resources necessary to sustain life (such as income, employment, housing, land, working conditions and livelihoods). The fourth dimension is the cultural dimension where we consider the patterns of relational exclusion that have been found to have cultural and historical origins, where people uphold norms and values which lead them to set themselves above others based on a variety of attributes. However, boundaries between social and cultural dimensions are difficult to draw because social participation is highly connected to cultural aspects such as values and norms translated into current social practices. Therefore, variables are identified under a socio-cultural domain.

### Data

Ghana is divided into10 administrative regions which are subdivided into 170 districts. The study uses data collected from 4050 representative households in five districts in Ghana covering the 3 ecological zones (coastal, forest and savannah). The household survey was conducted using Enumeration Areas (EAs) based on the 2000 Ghana Population and Housing Census for the selected districts. The five districts comprised Abura-Asebu- Kwamamkese in the Central Region, Kwaebibrim in the Eastern Region and Ejisu-Juaben Municipal in the Ashanti Region, Asutifi in the Brong Ahgafo Region and Savelugu-Nanton in the Northern Region. These EAs are made up of rural and urban localities and are determined by the Ghana Statistical Service (GSS) for nationally representative surveys. In each district, 27 EAs were selected by the GSS. After the listing to obtain the sampling frame, 30 households (with an additional 10 households for replacement) were systematically sampled for the interviews. Thus, in each district, 810 households (i.e. 30 households x 27 EAs) were interviewed resulting in a total of 4050 households with an estimated household population of 16,200.

In each household, the respondent was the head or an adult member who is normally responsible for major household decisions. Data on health services utilisation was collected using a disaggregated classification of health providers (Regional hospital, District hospital, Private/NGO hospital, Public health centre, Private clinic, Mission/NGO clinic, Private pharmacy, License chemical store) of outpatient care. In this paper, only utilisation of outpatient services at formal providers is considered. The recall period for outpatient visits was 2 weeks.

### Dependent and Independent variables

A range of patient characteristics determines whether patients are willing and able to make treatment choices. Some of these choices may also be influenced by social and cultural factors [13–15]. There is a large volume of literature which indicates that wealth and income affect treatment seeking behaviour especially in accessing formal health facilities [13, 16–18]. Beyond providing financial protection from the economic consequences of illness, health insurance is meant to improve access to healthcare [19, 20].

The dependent variable is a binary variable reflecting the use of formal healthcare (i.e. Regional hospitals, District hospitals and Public health centres). Informal care includes all individuals who did not seek care from formal healthcare providers. Among the independent variables are individual and household characteristics. Individual characteristics include age, gender, education, health insurance status, nature of illness. The SPEC variables include marital status (single or married), social networks (belonging to a social group or not) which fall under the sociocultural category. Household characteristics include a household welfare index as a proxy for household income. This is considered under the economic category. Five variables were created with the fifth quintile (highest income group) used as the base group (the omitted variable). The political category includes distance to health facility (irrespective of mode of transport).

### Regression models

Our basic regression model for determinants of utilisation can be defined as:

$$ Use{r}_i={\upbeta}_0+\kern0.5em {V}_{\mathrm{i}}{\upbeta}_1+{X}_{\mathrm{i}1}\upalpha +{\upvarepsilon}_{\mathrm{i}1} $$

Where *i = 1…n* represents individuals. *User*_*i*_*i*s a binary variable that denotes whether the individual used formal care or not. *Vi* is a set of SPEC variables (as described in Table 1), *Xi* is a set of remaining variables that may determine utilisation, and *εi* captures the random shock. Three logistic regression models are estimated. First, we estimated a simple regression model (Model 1) with only *Xi* variables, we then ran the regression with all variables—*Xi* and *Vi* variables in Model 2, and in the third model (Model 3) we included *Xi* variables and the SPEC variables.

**Table 1:** Description of variables in estimation

Dependent variable	Variable Abbreviation	Mean	Std. Dev.	*N*
User		0.914	0.280	1349
Independent variables				
*Individual characteristics*				
age = <18 years	<18 YEARS	0.477	0.499	16,124
18–69^a^		0.485	0.500	16,124
70 years and above	>70 YEARS	0.038	0.190	16,124
Male	MALE	0.466	0.499	16,178
Female^a^		0.534	0.499	16,178
No education^a^	NO EDUC	0.362	0.481	9219
Some primary	PRIMARY EDUC	0.183	0.387	9219
JSS/Middle	JSS EDUC	0.342	0.474	9219
Secondary and above	SECONDARY EDUC	0.113	0.317	9219
Insured	HEALTH INSURA NCE	0.732	0.443	16,100
Uninsured^a^		0.268	0.443	16,100
Chronic^a^	CHRONIC	0.037	0.189	15,991
Travel time to facility^b^				
District Hospital:				
Less than 15 mins.		0.189	0.419	16,120
15 to 60 mins.		0.593	0.513	16,120
above 60 mins.^a^		0.226	0.444	16,120
Regional Hospital:				
Less than 15 mins.		0.033	0.419	16,039
15 to 60 mins.		0.403	0.620	16,039
above 60 mins.^a^		0.616	0.617	16,039
SPEC variables				
Sociocultural (SC)				
Single		0.436	0.496	9392
No_association		0.611	0.529	5292
Political (P)				
Political_participation		0.909	0.288	5287
Economic (E)				
Wealth Quintile:				
First		0.205	0.403	13,690
Second		0.202	0.401	13,690
Middle		0.226	0.418	13,690
Fourth		0.179	0.383	13,690
Fifth^a^		0.189	0.391	13,690

Source: Household Survey, 2012

^a^Comparison group

^b^travel time irrespective of mode of transportation (in minutes)

#### Ethical considerations

Ethical clearance was sought and granted from the Institutional Review Board (IRB), of the Noguchi Memorial Institute for Medical Research (NMIMR), University of Ghana before the study was done. Study objectives, benefits, risks and the right to refuse participation and confidentiality of responses were explained to participants. Written informed consent was obtained from each participant.

## Results

### Description of the sample

In total, 16,200 individuals were available for the analysis in the survey data. Of these households, 73 % were insured, 53 % were female and 36 % had no education. Table 1 presents a description of variables in the estimation.

### Utilization of health services

Table 2 presents the percentage share of individual and household attributes of the users and non-users groups. A total of 1349 individuals reported seeking health care in the last 2 weeks. These users had a higher percentage of individuals with active insurance status (64 %) compared to 50 % of the non-users (*p* <0.00). We found that among the users, 53 % lived in urban areas; among the non-users 44 % lived in urban areas (*p* = 0.03). Also, among the users, 17 % were found to be in the lowest wealth quintile compared to 24 % among the non-users. Also, 30 % of non-users were more than one hour from the nearest district hospital compared to 19 % of users.

**Table 2:** Characteristics of users and non-users of healthcare services with reference to the recent reported illness/injury

	Status		*P*-value*
	Users	Non-users	
Characteristics	*N* = 1349	*N* = 14,851	
Sex			
Male	39.9	42.7	*P* = 0.000
Female	60.1	57.3	
Residence			
Urban	53.3	44.4	*P* = 0.026
Rural	46.7	55.6	
Age			
Children (≤18 years)	44.9	35.3	*P* = 0.314
Adult	47.1	54.3	
Elderly (≥70 years)	8.1	10.3	
Insurance status			
Active members	63.7	50.43	*P* = 0.000
Previous members	15.1	17.9	
No card	4.0	5.1	
Never insured	17.2	26.6	
% of adults (≥18 years) who ever attended school (*n* = 796)	65.1	73.0	
Mean years of schooling (≥6 years) (in years)	7.1	7.1	
Access to health (% of population who are more than 60 min from the nearest health facilities)			*P* = 0.15
Regional hospital	60.8	62.6	
District hospital	19.7	29.9	
Private/NGO hospital	32.0	33.7	
Public health centre	3.3	7.9	
Private clinic	23.4	28.7	
Mission/NGO clinic	29.4	28.7	
Private pharmacy	17.2	19.8	
License chemical store	2.3	1.7	
Access to transport and administrative infrastructure (mean time in minutes)			
The nearest tarmac road	19.0	27.3	*P* = 0.67
The nearest all-seasoned road	7.5	11.1	
Weekly market	25.3	28.8	
Daily market	15.5	21.3	
District capital	42.0	51.6	
The nearest place with daily bus /taxi services	9.5	10.0	
Wealth quintile (economic resources)			
First	17.2	23.9	*P* = 0.141
Second	21.9	17.1	
Middle	21.3	23.9	
Fourth	21.0	17.1	
Highest	18.5	18.0	

Source: Household Survey, 2012

* Chi-square test

### Effects of individual characteristics and SPEC variables on choice of health facility

Results from the logistic regressions models, are presented in Table 3. The models were run for respondants who indicated that they were ill and sought treatment for their illness within the 2 weeks preceding the date of the interview. This therefore affected the sample size since most of the respondents indicated that they there were not ill during that period. The dependent variable is a dichotomous variable where 1 means a person used formal means of treatment and 0 means the person chose traditional treatment option. The results in Models 1 and 2 show the marginal effects of the individual characteristics and the SPEC variables respectively on the dependent variable whereas the results in Model 3 show the combined effects of both the individual characteristics and the SPEC variables on the dependent variable.

**Table 3:** Estimation of Models (1–3) showing the Probability of Usage of Healthcare in last 2 weeks

Variables	Marginal Effects		
Dependent variable: Use of Formal facility	*Model 1*	*Model 2*	*Model 3*
age 18–69	−0.027		−0.007
Age 70 and above	0.012		0.025
Male	−0.066***	−0.055*	−0.066***
Some Primary	0.036		0.040*
JSS/Middle school	0.041*		0.048*
Secondary and above	0.094***		0.097***
Insured	0.068**		0.061*
Chronic	0.016		0.007
Travel Time to Facility:			
*District hospital*			
Less than 15 min	0.030		0.031
15 to 60 min	0.011		0.011
*Regional hospital*			
Less than 15 min	−0.242		−0.283
15 to 60 min	−0.040		−0.043*
SPEC Variables			
No association		−0.034	#
Single		−0. 011	−0.029
Wealth Quintile: First		0.051**	0.043
Second		#	#
Third		#	#
Fourth		0.027	−0.004
Number of Observations	667	607	645
LR Chi2	35.32	11.93	39.21
Prob > Chi2	0.000	0.036	0.000
Pseudo R-Squared	0.081	0.031	0.090

Notes: # Not enough observations

*p < 0.1

**p < 0.05

***p < 0.01

The regression results in Model 1 indicate that males have a lower probability (6.6 % lower) of using formal health facility than their female counterparts. The results in the Model 1 also depicts that the probability of using formal health facility for treatment increases with the level of education. Individuals with JSS/Middle school education are more likely (with a higher probability of 0.041) to use formal treatment facility relative to those with no education. Similarly, those with secondary education and above are most likely (9.4 % more likely) to seek treatment from a formal healthcare facility compared to those with no education.

The results in Model 2 also show that males are less likely to use formal treatment options relative to females. The results also depict that those in the first wealth quintile are more likely to use formal treatment options than those in the fifth wealth quintile. Similarly, the result in Model 3 shows that males are less likely to use formal treatment options than females. The results also confirm that the significance and the probability of using formal treatment option increases with the level of education (with no education being the comparison group). Those with health insurance coverage are still more likely to formal healthcare compared to those with no health insurance coverage. However, individuals who are 15 to 60 min away from the regional hospital are less likely (0.043 probability lower) to use formal treatment option.

## Discussion

This paper seeks to investigate factors that affect the utilisation of public and private outpatient healthcare services in Ghana. Our results indicate that, health insurance status, education and gender have been shown to be the three main determinants. A large proportion of the insured who reported ill, sought care from formal health care providers compared to the uninsured. This finding is similar to the results of other studies [18, 21–25]. Education and quite specifically, having secondary education and above is significant determinant of choice of care. An educated person is enlightened about the dangers and the benefits associated with the traditional and formal treatment options respectively and therefore would opt for the formal treatment options, all other things being equal. This supports the social exclusion theory that explains that the causes of inequality are based on the unequal structures of social systems [12]. Invariably, the better educated stand to gain more access to health care services as higher education allows for greater access to information and knowledge of the NHIS and its benefits.

The study shows that more women used formal healthcare services compared to men. There are a number of possible reasons why this could be the case. Fewer men are enrolled in the NHIS according to the results of this study which could explain why the numbers are skewed in favour of women. Alternatively, men who could not afford to pay the premiums of all household members would prefer for the women and children in the households to be insured. Are men voluntarily excluding themselves from the NHIS or this a consequence of other factors? Or do men prefer to seek care outside formal health care facilities? A plausible explanation could be that males in Ghana could be less concerned about their health and may also view the continuous attendance to the hospital as a sign of weakness. They may also resort to self-medication/ home treatment at the initial stages of sickness turning to formal treatment options when sickness aggravates or home treatment becomes ineffective. Although answers to these questions were beyond the scope of this study, this finding is intriguing and needs more attention as the consequences of males increasingly opting out of the health insurance scheme may have far reaching consequences for their future health.

Although we did not seek to understand why individuals were not enrolled in the scheme, one of the enabling factors we flagged was wealth status. Previous studies in Ghana have shown that individuals from richer quintiles are more likely to be enrolled into the NHIS scheme than those in poorer quintiles [8–10]. However, even if those in the highest quintiles are more likely to enroll, the results of this study show quite the opposite when it comes to utilisation of care. In our Model 2, where we consider only the SPEC variables, the individuals in the lowest quintile are more likely to seek care from formal healthcare services compared to individuals in the highest quintile. When we control for all other variables (Model 3) wealth becomes an insignificant determinant of utilisation of care. A potential explanation could be that the wealthier groups were not seeking care from NHIS accredited facilities. This is quite likely given evidence from studies in Ghana that indicate the insured receive less than optimum care at healthcare facilities [26–28].

This study has produced some interesting findings but it is not without a number of limitations. First, we are unable to draw ‘causal’ relationships between the dependent and independent variables due to the cross-sectional nature of the data. Also the independent variables may not capture the complete range of sociocultural, economic and political variables as explained in the SPEC methodology. Finally, restricting the sample to the use of outpatient services may not accurately assess the impact of the NHIS on utilisation and may overstate the extent to which the insured use health services. However, our findings are reflective of previous and more recent findings which suggest that utilisation of health services has been on the rise since the introduction of the NHIS in Ghana.

## Conclusions

We analyse the factors which determine choice of care in the framework of social protection, bedrock of Ghana’s health insurance scheme. The results indicate that health insurance, gender and education are significant determinants of healthcare utilisation. Compared to the uninsured, the insured are more likely to choose formal health facilities than informal care which confirms our initial hypothesis and also the results of other studies conducted on the NHIS in Ghana. However, several other factors may explain these findings.

In theory, the NHIS can be an effective system which provides the use to health care services that is affordable, available and offers financial protection in times of illness. Yet, equity concerns about the NHIS have been raised and the ability of the NHIS to ensure equitable access for vulnerable groups has received attention over the years. The voluntary nature of the scheme even though there are efforts to make the scheme mandatory, means that the risk pool has been narrowed mainly to the poor and sick with the exception of those whose contributions to the scheme are automatically deducted at source. Regardless of, gender, wealth status, or geography, efforts must be made to encourage more people to enroll in the scheme in order to avoid inequities. Greater support from families, friends and communities is linked to better health and hence the relevance of social networks in the utilisation of care. Social networks provide the necessary channels for the dissemination of information some of which may enable individuals to hear about the NHIS; be encouraged to enroll in it and therefore gain access to healthcare services.

### Availability of data and materials

The data is not publically available. The study uses primary data which was conducted as part of an EC grant and the following disclaimers have been added in the paper

- The disclaimer on funding—‘The funder was not involved in the research and preparation of the article, including study design; collection, analysis and interpretation of data; writing of the article; nor in the decision to submit it for publication’.
- The ethical approval—‘Ethical clearance was sought and granted from the Institutional Review Board (IRB), of the Noguchi Memorial Institute for Medical Research (NMIMR), University of Ghana before the study was done. Study objectives, benefits, risks and the right to refuse participation and confidentiality of responses were explained to participants. Written informed consent was obtained from each participant.’

## Abbreviations

CHPS: Community Health Posts Services
EAs: Enumeration Areas
IRB: Institutional Review Board
NGOs: Non-Governmental Organisations
NHIS: National Health Insurance Scheme
NMIMR: Noguchi Memorial Institute for Medical Research
SEKN: Social Exclusion Knowledge Network
SPEC: social, political, economic and cultural

## References

[1] Dror D, Jacquier C (1999). Micro-insurance: extending health insurance to the excluded. Int Soc Secur Rev.

[2] Preker A, Carrin G, Dror DM (2002). Effectiveness of community health financing in meeting the cost of illness. Bull World Health Organ.

[3] Nyman JA (2003). The Theory of Demand for Health Insurance.

[4] Ekman B (2004). Community-based health insurance in low-income countries: a systematic review of the evidence. Health Policy Plan.

[5] Carrin G, Waelkens MP, Criel B (2005). Community-based health insurance in developing countries: a study of its contribution to the performance of health financing systems. Trop Med Int Health.

[6] Ghana Health Service (2008). Annual Report 2007. Ghana Health Service, Ghana. Available at: http://www.ghanahealthservice.org/ghs-item-list.php?scid=52. Accessed 12 Feb 2014.

[7] Dixon J, Tenkorang EY, Luginaah I (2011). Ghana’s National Health Insurance Scheme: helping the poor or leaving them behind?. Environ Plann-Part C.

[8] Jehu-Appiah C, Aryeetey G, Spaan E, De Hoop T, Agyepong I, Baltussen R (2011). Equity aspects of the National Health Insurance Scheme in Ghana: Who is enrolling, who is not and why?. Soc Sci Med.

[9] Chankova S, Atim C, Hatt L, Escobar M-L, Griffin C, Shaw RP (2010). Ghana’s National Health Insurance Scheme. The impact of health insurance in low- and middle-income countries.

[10] Parmar D, Williams G, Dkhimi F, Ndiaye A, Asante FA, Arhinful DK, Mladovsky P (2014). Enrolment of older people in social health protection programs in West Africa –Does social exclusion play a part?. Soc Sci Med.

[11] National Health Insurance Authority (2012). National Health Insurance Authority Status Report: Operations, December 2012.

[12] SEKN (2008). Understanding and tackling social exclusion. Final report to the WHO Commission on Social Determinants of Health from the Social Exclusion Knowledge Network.

[13] Sahn D, Stifel D (2003). Exploring alternative measures of welfare in the absence of expenditure data. Rev Income Wealth.

[14] Lindelow M (2005). The utilisation of curative healthcare in Mozambique: does income matter?. J Afr Econ.

[15] Amin R, Shah NM, Becker S (2010). Socioeconomic factors differentiating maternal and child health-seeking behaviour in rural Bangladesh: A cross-sectional analysis. Int J Equity Health.

[16] Akin JS, Guilkey D, Denton H (1995). Quality of services and demand for health care in Nigeria: a multinomial probit estimation. Soc Sci Med.

[17] Gertler P, van der Gaag J (1990). The Willingness to Pay for Medical Care: Evidence from Two Developing Countries.

[18] Fenny AP, Asante FA, Enemark U, Hansen KS (2015). Treatment-Seeking Behaviour and Social Health Insurance in Africa: the Case of Ghana Under the National Health Insurance Scheme. Global J Health Sci.

[19] Nyman JA (1999). The value of health insurance: the access motive. J Health Econ.

[20] Hsia J, Kemper E, Kiefe C, Sofaer S, Bowen D, Kiefe CI, Zapka J, Mason E, Lillington L, Limacher M (2000). The importance of health insurance as a determinant of cancer screening: evidence from the Women’s Health Initiative. Prev Med.

[21] Danso-Appiah A, Stolk WA, Bosompem KM, Otchere J, Looman CW, Habbema JD, de Vlas SJ (2010). Health seeking behaviour and utilization of health facilities for schistosomiasis-related symptoms in Ghana. PLoS Neg Trop Dis.

[22] Blanchet NJ, Fink G, Osei-Akoto I (2012). The effect of Ghana’s National Health Insurance Scheme on health care utilisation. Ghana Med J.

[23] Mensah J, Oppong JR, Schmidt CM (2010). Ghana's National Health Insurance Scheme in the context of the health MDGs: An empirical evaluation using propensity score matching. Health Econ.

[24] Brugiavini A, Pace N (2015). Extending health insurance in Ghana: effects of the National Health Insurance Scheme on maternity care. Health Econ Rev.

[25] Alhassan RK, Nketiah-Amponsah E, Akazili J, Spieker N, Arhinful DK, Rinke de Wit TF (2015). Efficiency of private and public primary health facilities accredited by the National Health Insurance Authority in Ghana. Cost Eff Resour Alloc.

[26] Dixon J, Tenkorang EY, Luginaah I (2013). Ghana’s National Health Insurance Scheme: a national level investigation of members’ perceptions of service provision. BMC Int Health Hum Rights.

[27] Fenny AP, Enemark U, Asante FA, Hansen KS (2014). Patient satisfaction with primary health care – A Comparison between the Insured and Non-Insured under the National Health Insurance Policy in Ghana. Global J Health Sci.

[28] Boateng D, Awunyor-Vitor D (2013). Health insurance in Ghana: evaluation of policy holders’ perceptions and factors influencing policy renewal in the Volta region. Int J Equity Health.

